# Prodigious plant methylomes

**DOI:** 10.1186/s13059-016-1065-2

**Published:** 2016-09-27

**Authors:** Mary Gehring

**Affiliations:** Whitehead Institute for Biomedical Research, Nine Cambridge Center, Cambridge, MA 02142 USA

## Abstract

Schmitz and colleagues recently investigated DNA methylation patterns in diverse flowering plant species, finding substantial variation in the extent and distribution of methylation in angiosperms.

Please see related Research article: http://www.doi.org/10.1186/s13059-016-1059-0

## Introduction

For the past decade, plant research has improved our understanding of the distribution and function of eukaryotic DNA methylation at a genome-wide scale. Significant progress has been made in understanding DNA methylation in the model plant *Arabidopsis thaliana* and more recently in maize, two species that diverged at the monocot–eudicot split approximately 150 million years ago. In their recent article, Robert Schmitz and colleagues analyzed whole-genome bisulfite sequencing data from 34 diverse flowering plant species to vastly expand our knowledge of plant methylome patterns [1]. Results from their study showed substantial variation in the extent and distribution of DNA methylation in angiosperms.

## Extensive methylation in plant genomes

**Fig. 1:**
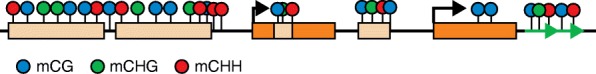
Generic methylation patterns in plants. Genes (*dark orange boxes*) are CG methylated (*blue dots*) but can also contain non-CG methylation (*green* and *red dots*) if TEs or TE fragments (*light orange boxes*) are present internally. TEs are methylated in all sequence contexts, with CHH methylation more concentrated at TE ends than internal regions in some species. Other sequence features, such as direct repeats (*green arrows*), are also subject to DNA methylation in all sequence contexts

Plants have relatively high concentrations of 5-methylcytosine (5mC) compared to non-plant species [2]. This is because plants have DNA methylation in all three sequence contexts—CG, CHG, and CHH (where H is any base besides G)—with separate methylation pathways responsible for each sequence context. Generally, CG methylation is found in transcribed gene regions, whereas both CG and non-CG methylation is associated with transposable elements (TEs) and repeats (Fig. 1). The dual presence of CG and non-CG methylation is often associated with transcriptional silencing, although there are many exceptions to this.

DNA methylation is established by chromomethyltransferase (CMT) enzymes and the de novo methyltransferase DRM2. CMT enzymes interact with methylated histone H3K9, a mechanism by which repressive histone methylation and DNA methylation reinforce one another [3]. DRM2 is directed to sites of action by 24-nucleotide small RNAs in a process known as RNA-directed DNA methylation (RdDM) [4]. CG methylation is maintained after DNA replication by the maintenance methyltransferase MET1 and CHG methylation is maintained by CMTs. CHH methylation must be constantly established; by definition a methylated C in a CHH site does not have a corresponding C on the antiparallel DNA strand.

## Characteristics of flowering plant methylomes

Single-base resolution methylomes were first described for *Arabidopsis*. This model organism continues to be essential for deciphering the genetic players that underlie methylation patterning and function. Methylation research in maize has revealed some features that differ from *Arabidopsis*. For example, there is a high concentration of 100-bp regions with >25 % CHH methylation in gene flanking regions, known as CHH islands, which may protect silent TEs from the activity of adjacent genes [5]. *Arabidopsis* has a compact genome with relatively few TEs; in contrast, maize has a very large genome with 85–90 % TEs. It is unclear whether some of the unique methylation features observed in these species are truly unique or if they instead represent insufficient sampling across the flowering plant phylogenetic tree.

Armed with whole genome bisulfite sequencing data from leaf tissue of eight previously studied species, as well as 26 newly generated flowering plant methylomes, Schmitz and colleagues analyzed the distribution and extent of CG, CHG, and CHH methylation in relation to TE content, gene expression, and genome size [1]. Many of the findings from this study are consistent with previous data from *Arabidopsis*, maize, rice, and poplar: (1) methylation is highest in the CG context and lowest in CHH, which reflects the different mechanisms by which these types of methylation are maintained; (2) repeats are highly methylated in the CG context; (3) gene bodies with typical CG methylation patterns are moderately expressed; and (4) CG methylation concentrated around the transcriptional start site is associated with repressed transcription.

## Widespread methylome variation

Results from the large-scale methylome analysis by Schmitz and colleagues also demonstrated some surprising patterns. For example, results showed that maize is not an extreme example of a highly methylated genome. Beet (*Beta vulgaris*) has higher methylation levels than any of the other species assayed, with particularly high CHH methylation, seemingly driven by a high percentage of genes that contain repetitive elements. Among repeats, there was substantial interspecies variation in the amount of CHG and CHH methylation, and only CHG methylation correlated with genome size across all species. CHH islands in gene flanking regions were not restricted to maize and were found in many other species. Yet, the positive correlation between CHH islands and gene expression in maize was not universal. It remains unclear if all regions annotated as CHH islands are comparable across- or even within-species, owing to the fairly broad definition of CHH islands.

Schmitz and colleagues analyzed multiple species from the same family, a powerful aspect of the study that allowed broader phylogenetic conclusions to be drawn. For example, *Arabidopsis* has lower CG methylation than any of the other examined species but that reduced methylation is not restricted to *Arabidopsis*. The six examined species of the Brassicaceae family, of which *Arabidopsis* is a member, have distinctly lower levels of CHG and CHH methylation compared to other families. The grasses (Poaceae) have overall low levels of CHH methylation, particularly in the inner regions of repeats, but the CHH methylation that is present is concentrated at high levels in smaller regions of the genome.

What causes interspecies methylation variation? In some species there may be differences in the activity of, or mutations in, DNA methylation machinery. Schmitz and colleagues have shown previously that *Eutrema salsugineum*, which has the lowest levels of CHG methylation and no CG gene body methylation, lacks a functional CMT3 enzyme [6]. Genome-wide association studies in *Arabidopsis* have linked methylation variation to *CMT2* [7], which is absent in maize. Another potent contributor to interspecies methylation variation is likely to be genomic content, specifically the percentage of repetitive elements.

## Perspective

The study by Schmitz and colleagues provides several intriguing findings that warrant follow-up study. Outside of the grasses, multiple dicots (grape, cassava, wild strawberry, and others) also had low levels of CHH methylation, independent of genetic relatedness [1]. The authors speculate that low CHH methylation could be a result of how these species are propagated agriculturally, through clonal production. This hypothesis is intriguing in light of evidence that (1) CHH methylation is partially lost during male gametogenesis but is restored in the embryo [8] and (2) RdDM acts progressively during reproductive development over multiple generations, at least in genomes that have undergone massive hypomethylation [9]. A sexual reproductive phase may be essential to reinforce and preserve methylation patterning.

Results from the study also showed that conserved non-coding sequences, which are home to gene regulatory regions like transcription factor binding sites, are less methylated than expected. Schmitz and colleagues suggest that these regions remain methylation-free because DNA methylation might negatively impact transcription factor binding, as has been shown recently in vitro [10]. An alternative or additional explanation is that sequences free of methylation are more likely to be conserved because 5mC is subject to frequent spontaneous deamination and is inherently mutagenic.

It is not yet clear if there are functional consequences of the differences in methylation and, if so, what they might be. However, the data presented by Schmitz and colleagues generats many hypotheses for future investigation. The rules of DNA methylation are not yet fully written.

## Abbreviations

CMT2: Chromomethylase2
CMT3: Chromomethylase3
DRM2: Domains Rearranged Methyltransferase2
MET1: Methyltransferase1
